# Musculoskeletal pain and loneliness, social support and social engagement among older adults: Analysis of the Oxford Pain, Activity and Lifestyle cohort

**DOI:** 10.1002/msc.1526

**Published:** 2020-11-17

**Authors:** Philippa J. A. Nicolson, Esther Williamson, Alana Morris, Maria T. Sanchez‐Santos, Julie Bruce, Alan Silman, Sarah E. Lamb

**Affiliations:** ^1^ Nuffield Department of Orthopaedics, Rheumatology and Musculoskeletal Sciences (NDORMS) University of Oxford Botnar Research Centre Oxford UK; ^2^ Warwick Clinical Trials Unit Warwick Medical School University of Warwick Coventry UK; ^3^ College of Medicine and Health University of Exeter Exeter UK

**Keywords:** musculoskeletal, older people, psychological and social impact

## Abstract

**Background:**

Musculoskeletal (MSK) pain is common in older adults. Physical and psychological consequences of MSK pain have been established, but it is also important to consider the social impact. We aimed to estimate the association between MSK pain and loneliness, social support and social engagement.

**Methods:**

We used baseline data from the Oxford Pain, Activity and Lifestyle study. Participants were community‐dwelling adults aged 65 years or older from across England. Participants reported demographic information, MSK pain by body site, loneliness, social support and social engagement. We categorised pain by body regions affected (upper limb, lower limb and spinal). Widespread pain was defined as pain in all three regions. We used logistic regression models to estimate associations between distribution of pain and social factors, controlling for covariates.

**Results:**

Of the 4977 participants analysed, 4193 (84.2%) reported any MSK pain, and one‐quarter (*n* = 1298) reported widespread pain. Individuals reporting any pain were more likely to report loneliness (OR [odds ratio]: 1.62; 95% CI [confidence interval]: 1.32–1.97) or insufficient social support (OR: 1.54; 95% CI: 1.08–2.19) compared to those reporting no pain. Widespread pain had the strongest association with loneliness (OR: 1.94; 95% CI: 1.53–2.46) and insufficient social support (OR: 1.71; 95% CI: 1.14–2.54). Pain was not associated with social engagement.

**Conclusions:**

Older adults commonly report MSK pain, which is associated with loneliness and perceived insufficiency of social support. This finding highlights to clinicians and researchers the need to consider social implications of MSK pain in addition to physical and psychological consequences.

## 1 INTRODUCTION

Musculoskeletal (MSK) pain is highly prevalent among older adults (Thomas, Peat, Harris, Wilkie, & Croft, 2004). It is the leading cause of years lived with disability worldwide, and this burden is expected to continue to increase as the population ages (Vos et al., 2015). Not only are older adults more likely to report/experience any MSK pain, they are more likely than younger adults to experience pain at more than one bodily site, and more likely to experience widespread pain (Birrell, 2004; Carnes et al., 2007; Dragioti, Larsson, Bernfort, Levin, & Gerdle, 2017; Kamaleri, Natvig, Ihlebaek, & Bruusgaard, 2008).

Loneliness is the subjective feeling of being alone or separated from others (Cacioppo & Cacioppo, 2018). Social support commonly refers to support given to an individual from other people, and the degree of an individual's satisfaction with the support they receive (Gobbens, van Assen, Luijkx, Wijnen‐Sponselee, & Schols, 2010). Social engagement is the extent which an individual participates in social activities, commonly measured by membership of clubs, groups and societies (Bath & Deeg, 2005). Loneliness and social support represent an individual's perception of their social contacts and support, whereas social engagement is more representative of actual participation.

Loneliness, perceived insufficiency of social support and a lack of social engagement are particularly prevalent with increasing age, with up to 40% of adults aged 50 years or older reporting loneliness over 6 years of follow‐up in a large US cohort (Luo, Hawkley, Waite, & Cacioppo, 2012). Loneliness, perceived insufficiency of social support and a lack of social engagement have all been found to be associated with multiple adverse health‐related states among older adults, including increased risk of cognitive decline, frailty and mortality (Herrera‐Badilla, Navarrete‐Reyes, Amieva, & Avila‐Funes, 2015; Luo et al., 2012; Shankar, McMunn, Banks, & Steptoe, 2011).

The negative effects of single site, multisite and widespread MSK pain on physical and psychological outcomes are well established (Butera, Roff, Buford, & Cruz‐Almeida, 2019). However, these findings do not consider the wider implications of MSK pain on important social determinants of healthy ageing (Makizako et al., 2015).

Given the impact MSK pain has on the health and well‐being of older adults, and the negative health consequences of poor social functioning, it is important to understand the relationship between these factors. Previous studies have focused on the relationship between the presence of any MSK pain and social factors (Emerson, Boggero, Ostir, & Jayawardhana, 2018; Smith, Dainty, Williamson, & Martin, 2019). However, different regional distributions of MSK pain are likely to differentially impact on outcomes. A better understanding of the effect of regional and widespread MSK pain on social factors may allow treatments to be appropriately tailored for maximal benefit to the individual.

The objectives of this study were twofold as follows: (1) to identify whether any, regional and widespread MSK pain are associated with loneliness, social support and social engagement, and (2) to determine whether the distribution of MSK pain is associated with increased loneliness, lack of social support and poor social engagement among older adults, after adjusting for other possible determinants.

## 2 METHODS

Data for this cross‐sectional analysis were identified from baseline responses in the Oxford Pain, Activity and Lifestyle (OPAL) study collected between October 2016 and September 2018 (Sanchez Santos et al., 2020). Ethical approval was provided by the London Brent Research Ethics Committee (16/LO/0348).

### 2.1 Participants

The OPAL study is a prospective longitudinal cohort study of community‐dwelling older adults aged 65 years or older recruited from 35 general practices across England. A detailed profile of the cohort is published elsewhere (Sanchez Santos et al., 2020). Eligible participants were identified from electronic record searches of primary care practice lists which identified a random sample of up to 400 patients per practice (median: 365; range 158–400) for invitation, stratified into two age bands (65–74 and 75 years and over). Individuals were ineligible if they lived in residential care or a nursing home, those with known terminal illness with a life expectancy of less than 6 months, those who presented with severe health or social concerns sufficient to preclude approach, or those considered unable to provide informed consent.

A total of 12,839 patients were contacted by their general practice and invited to take part in the OPAL study. A consent form, patient information leaflet and baseline questionnaire were sent. People who did not return the questionnaire were sent one postal reminder 4 weeks after the original invitation. Among invited participants, 42.1% (*N* = 5409) returned the baseline questionnaire and were enrolled in the study.

Participants were eligible for this analysis if they returned baseline questionnaires with no missing data for the pre‐specified variables analysed.

### 2.2 Measures

#### 2.2.1 Musculoskeletal pain

We identified MSK pain sites using the Nordic Musculoskeletal Questionnaire (Kuorinka et al., 1987). We categorised the nine named pain sites into three bodily regions: upper limb (shoulders, elbows, wrist or hands); spinal (neck, upper back and lower back) and lower limb (hips, knees ankles or feet). We defined regional pain as any pain in one of these bodily regions. The American College of Rheumatology (ACR) defined widespread pain as pain above and below the waist, pain on the right and left sides of the body, or axial skeletal pain (Wolfe et al., 2010). We modified ACR criteria to classify widespread pain as pain reported in all three bodily regions (upper limb, lower limb and spinal). We could not include the ACR criteria for contralateral pain because the OPAL study data did not include this characteristic.

#### 2.2.2 Social factors

We assessed loneliness and perceived sufficiency of social support using two questions within the social domain of the Tilburg Frailty Indicator: (Gobbens et al., 2010) ‘Do you miss having other people around you?’ (loneliness: sometimes/yes/no) and ‘Do you receive enough support from other people?’ (social support: yes/no). ‘Sometimes’ and ‘Yes’ responses were combined to create a dichotomous outcome. Each of these questions have been found to correlate positively and significantly with established longer measures of the construct they assess (the Loneliness Scale and the Social Support List; Gobbens et al., 2010). We assessed social engagement by asking about membership of the following organisations, clubs or societies: political party, trade union or environmental groups; tenants or residents' groups or Neighbourhood Watch; church or other religious groups; charitable associations; education, arts or music groups or evening classes; social clubs; sports clubs, gyms, exercise classes, or any other organisations, clubs or societies. Respondents selected all options that applied or ‘No, I am not a member of any organisations, clubs or societies’. We dichotomised social engagement as no membership or membership of one or more clubs/societies.

#### 2.2.3 Participant characteristics

Demographic information collected included self‐reported age, sex, height, weight, smoking status (ever/never) and living arrangement (alone/with others). We calculated Body Mass Index (BMI) by dividing weight in kilogrammes by height in metres squared. Socioeconomic factors assessed included self‐reported education level (school/higher education), physical demands of their main occupation before retirement (very light‐light; moderate; strenuous‐very strenuous) and the UK Index of Multiple Deprivation (IMD, derived from individual respondent postcode). The IMD provides a relative measure of deprivation based on seven domains: income; employment; education, skills and training; health and disability; crime; barriers to housing and services, and living environment (Smith et al., 2015). For this analysis, IMD scores were divided into quintiles from 1 = 20% most deprived to 5 = 20% least deprived in England.

Participants were asked to report chronic health conditions from a predetermined list of doctor‐diagnosed conditions, including arthritis, angina or heart troubles, cancer, chronic lung disease, dementia, diabetes, high blood pressure, osteoporosis, Parkinson's disease, peripheral vascular disease or stroke. We categorised the number of health conditions reported as: 0; 1 or 2 or ≥3. We assessed pain severity using item 4 of the EQ‐5D‐5L (Herdman et al., 2011). The five‐level responses for pain were categorised as ‘no/slight pain’, ‘moderate pain’ or ‘severe/extreme pain’. We assessed mobility using item 1 of the EQ‐5D‐5L (Herdman et al., 2011). The five‐level responses for mobility were categorised as ‘no/slight problems walking’, ‘moderate problems walking’ or ‘severe problems/unable to walk’.

### 2.3 Statistical analysis

We analysed data in Stata Statistical Software Release 15.0 (StataCorp LP) and report the study in accordance with the STrengthening the Reporting of OBservational studies in Epidemiology recommendations (Vandenbroucke et al., 2014). We calculated prevalence of any MSK pain, regional and widespread pain for the total sample and stratified by the two age bands recruited (65–74 and 75 years and over) and sex. Demographic and health‐related characteristics were described by any pain, by region and number of regions of MSK pain. We used unadjusted and adjusted logistic regression models to estimate the association between regional and widespread pain and loneliness, social support and social engagement. Models were adjusted for the following covariates: age, sex (reference category: males), BMI, smoking status (reference category: never), living alone (reference category: living with others), education level (reference category: higher education), physical demands of occupation (reference category: very light/light), index of multiple deprivation (reference category: 20% least deprived), number of health conditions (reference category: 0), pain severity (reference category: no/slight pain) and mobility limitations (reference category: no/slight problems).

## 3 RESULTS

### 3.1 Characteristics of included participants

**FIGURE 1:**
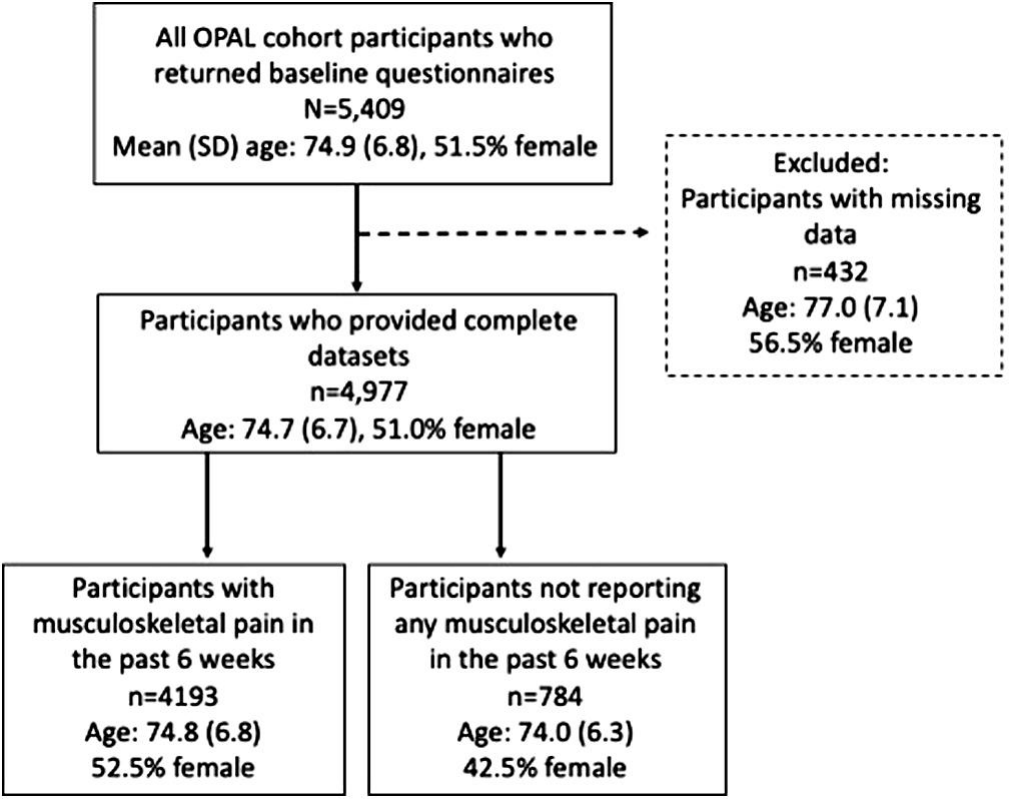
Flow‐chart illustrating analysed study participants contributing to analysis from the recruited Oxford Pain, Activity and Lifestyle (OPAL) cohort

Of the 5409 participants enrolled in the OPAL cohort, 432 (7.9%) were excluded from this analysis because of missing data in one or more of the variables analysed (Figure 1). Thus, 4977 (92.1%) participants were included; 2832 aged 65 to 74 years and 2145 aged 75 years and over. The majority of excluded participants had missing data for one variable only (381/432; 88.2%), most commonly BMI (224/432; 51.9%). Of the included participants 2540 (51.0%) were female. Participants excluded from the analysis due to missing data (*n* = 432) were older (mean [SD; standard deviation] age: 77.0 [7.1] vs. 74.7 [6.7]), more likely to be female (56.5% vs. 51.0%), live alone (36.3% vs. 28.3%), have completed school education only (69.8% vs. 64.0%) and report moderate or severe mobility limitations (23.4% vs. 19.0%) compared to those included in the analysis (Table A1). The items with the highest rates of data missingness were height or weight (224/432) or social support (100/432).

### 3.2 Characteristics of participants reporting any MSK pain

A total of 4193/4977 (84.3%) participants reported pain at one or more of the named body sites (Table 1). The most commonly reported sites of pain were low back pain (1218/2540; 48.0% of women; 1030/2437; 42.3% of men) and knee pain (1032/2540; 40.6% of women; 864/2437; 35.5% of men), followed by wrist or hand pain among women (966/2540; 38.0%) and neck pain among men (630/2437; 25.9%). Compared to those who reported no MSK pain, participants reporting any pain were more likely to be female (52.6% vs. 42.5%), report three or more health conditions (26.2% vs. 6.8%), and report moderate or severe mobility limitations (21.7% vs. 4.0%; Table 2). Participants with any MSK pain more commonly reported loneliness compared to those with no pain (39.1% vs. 24.2%; Table 3).

**TABLE 1:** Musculoskeletal pain sites and regions by age category and gender

	Total (*n* = 4977)	65–74 years		75 years+	
		Female (*n* = 1436)	Male (*n* = 1396)	Female (*n* = 1104)	Male (*n* = 1041)
Bodily sites reported as painful *n* (%)					
Shoulder/s	1485 (29.8)	458 (31.9)	359 (25.7)	409 (37.91)	259 (24.9)
Elbow/s	305 (6.1)	94 (6.6)	97 (7.0)	63 (5.7)	52 (4.9)
Wrist/Hand/s	1575 (31.7)	531 (37.0)	356 (25.5)	435 (39.4)	253 (24.3)
Neck	1514 (30.4)	483 (33.6)	355 (25.4)	401 (36.3)	275 (26.4)
Upper back	470 (9.4)	163 (11.4)	80 (5.7)	155 (14.0)	72 (6.9)
Low back	2248 (45.2)	669 (46.6)	590 (42.3)	549 (49.7)	440 (42.3)
Hip/s	1365 (27.4)	448 (31.2)	305 (21.9)	357 (32.3)	255 (24.5)
Knee/s	1896 (38.1)	555 (38.7)	465 (33.3)	477 (43.2)	399 (38.3)
Ankles/Feet	1213 (24.4)	366 (25.5)	291 (20.9)	324 (29.4)	232 (22.3)
Regional pain *n* (%)					
No MSK pain reported	784 (15.8)	206 (14.4)	279 (20.0)	127 (11.5)	172 (16.5)
Any MSK pain	4193 (84.3)	1230 (85.7)	1117 (80.0)	977 (88.5)	869 (83.5)
Pain in one region	1480 (29.7)	386 (26.09)	466 (33.4)	280 (25.4)	348 (33.4)
Pain in two regions	1415 (28.4)	416 (29.0)	368 (26.4)	327 (29.6)	304 (29.2)
Pain in three regions (widespread)	1298 (26.1)	428 (29.8)	283 (20.3)	370 (33.5)	217 (20.9)

Abbreviation: MSK, musculoskeletal.

**TABLE 2:** Characteristics of participants by number of bodily regions of musculoskeletal pain

	Total (*n* = 4977)	No MSK pain (*n* = 784)	Any MSK pain (*n* = 4193)	Pain in one region (*n* = 1480)	Pain in two regions (*n* = 1415)	Widespread pain (*n* = 1298)
Age, mean (SD) years	74.7 (6.7)	74.0 (6.3)	74.9 (6.8)	74.6 (6.6)	74.9 (6.8)	75.1 (6.9)
Sex, female (%)	2540 (51.0)	333 (42.5)	2207 (52.6)	666 (45.0)	743 (52.5)	798 (61.5)
BMI, mean (SD) kg/m^2^	26.6 (4.9)	25.6 (4.0)	26.8 (5.0)	26.2 (4.7)	26.6 (4.7)	27.7 (5.5)
Living alone, *n* (%)	1408 (28.3)	198 (25.3)	1210 (28.9)	388 (26.2)	402 (28.4)	420 (32.4)
School education only, *n* (%)	3184 (64.0)	489 (62.4)	2695 (64.3)	900 (60.8)	909 (64.2)	862 (68.3)
Index of Multiple Deprivation						
5 (20% least deprived)	1738 (34.9)	302 (38.5)	1436 (34.3)	515 (34.8)	512 (36.2)	409 (31.5)
4	1080 (21.7)	165 (21.1)	915 (21.8)	353 (23.9)	283 (20.0)	279 (21.5)
3	1067 (21.4)	153 (19.5)	914 (21.8)	339 (22.9)	291 (20.6)	284 (21.9)
2	590 (11.9)	85 (10.8)	505 (12.0)	157 (10.6)	191 (13.5)	157 (12.1)
1 (20% most deprived)	502 (10.1)	79 (10.1)	423 (10.1)	116 (7.8)	138 (9.8)	169 (13.0)
Number of health conditions						
0	854 (17.2)	285 (36.4)	569 (13.6)	329 (22.2)	184 (13.0)	56 (4.3)
1–2	2971 (59.7)	446 (56.9)	2525 (60.2)	921 (62.2)	876 (61.9)	728 (56.1)
≥3	1152 (23.2)	53 (6.8)	1099 (26.2)	230 (15.5)	355 (25.1)	514 (39.6)
Pain severity (EQ‐5D‐5L)						
No/slight pain	3591 (72.2)	759 (96.8)	2832 (67.5)	1277 (86.3)	987 (69.8)	568 (43.8)
Moderate pain	1056 (21.2)	23 (2.9)	1033 (24.6)	182 (12.3)	359 (25.4)	492 (37.9)
Severe/extreme pain	330 (6.6)	2 (0.3)	328 (7.8)	21 (1.4)	69 (4.9)	238 (18.3)
Mobility limitations (EQ‐5D‐5L)						
No/slight problems walking	4034 (81.1)	753 (96.1)	3281 (78.3)	1337 (90.3)	1132 (80.0)	812 (62.6)
Moderate problems walking	643 (12.9)	22 (2.8)	621 (14.8)	107 (7.2)	213 (15.1)	301 (23.2)
Severe problems walking/Unable to	300 (6.0)	9 (1.2)	291 (6.9)	36 (2.4)	70 (5.0)	185 (14.3)

Abbreviations: BMI, Body Mass Index; MSK, musculoskeletal; SD, standard deviation.

**TABLE 3:** Social factor responses of participants by number of bodily regions of musculoskeletal pain; All values *n* (%)

	Total (*n* = 4977)	No MSK pain (*n* = 784)	Any MSK pain (*n* = 4193)	Pain in one region (*n* = 1480)	Pain in two regions (*n* = 1415)	Widespread pain (*n* = 1298)
Loneliness	1829 (36.8)	190 (24.2)	1639 (39.1)	496 (33.5)	526 (37.2)	617 (47.5)
Perceived insufficient social support	451 (9.1)	40 (5.1)	411 (9.8)	100 (6.8)	150 (10.6)	161 (12.4)
Not socially engaged (no club/society membership)	1502 (30.2)	237 (30.2)	1265 (30.2)	417 (28.2)	407 (28.8)	441 (34.0)

Abbreviation: MSK, musculoskeletal.

### 3.3 Characteristics of participants reporting regional MSK pain

A total of 2895/4977 (58.2%) participants reported MSK pain in one (*n* = 1480) or two regions (*n* = 1415; Table 1). Among those who reported pain in one region, this was most commonly the lower limb (*n* = 631; Table A2). Among those who reported pain in two regions lower limb and spinal pain was most common (*n* = 617).

### 3.4 Characteristics of participants reporting widespread MSK pain

One quarter of included participants (1298/4977; 26.1%) reported widespread MSK pain (Table 1). The proportion of participants reporting widespread pain increased with advancing age among women only (29.8% of women aged 65 to 74 vs. 33.5% of women aged 75+; 20.3% of men aged 65 to 74 vs. 20.9% of men aged 75+). Compared to those who reported no pain, those who reported widespread pain were more likely to be female (61.5% vs. 42.5%), live alone (32.4% vs. 25.3%) and live in the most deprived areas of England (13.0% vs. 10.1%; Table 2). Participants with widespread pain more frequently reported having three or more health conditions (39.6% vs. 6.8%) and were more likely to report moderate or severe mobility limitations (37.5% vs. 4.0%) compared to those who reported no pain. One‐fifth of participants with widespread pain reported severe or extreme pain severity (18.3%).

### 3.5 Association between any MSK pain and social factors

Participants who reported any MSK pain were more likely to report loneliness than those who reported no pain (OR [odds ratio]: 2.01; 95% CI [confidence interval]: 1.68–2.38), and this effect remained after adjusting for demographic factors, pain severity and mobility limitation (OR: 1.62; 95% CI 1.33–1.97; Table 4). Any MSK pain was also associated with insufficient social support compared to those who reported no pain in unadjusted (OR: 2.02; 95% CI: 1.45–2.82) and adjusted models (OR: 1.55; 95% CI 1.10–2.21). We found no significant association between the presence of any MSK pain and extent of self‐reported social engagement.

**TABLE 4:** Unadjusted and adjusted odds ratios (95% CI) for limitations in loneliness, social support and social engagement by number of regions of musculoskeletal pain

	Loneliness		Perceived insufficient social support		Not socially engaged	
	OR	95% CI	OR	95% CI	OR	95% CI
No MSK pain (*n* = 784)	1.00	Reference	1.00	Reference	1.00	Reference
Any MSK pain (*n* = 4193)						
Model 1	2.01	1.68–2.38	2.02	1.45–2.82	1.00	0.84–1.18
Model 2	1.62	1.33–1.97	1.55	1.10–2.21	0.83	0.69–1.00
Pain in one region (*n* = 1480)						
Model 1	1.58	1.30–1.92	1.34	0.92–1.97	0.91	0.75–1.09
Model 2	1.55	1.25–1.92	1.28	0.88–1.89	0.88	0.72–1.07
Pain in two regions (*n* = 1415)						
Model 1	1.85	1.52–2.25	2.20	1.53–3.16	0.93	0.77–1.13
Model 2	1.56	1.25–1.94	1.84	1.26–2.68	0.78	0.63–1.00
Widespread pain (*n* = 1298)						
Model 1	2.83	2.33–3.45	2.63	1.84–3.77	1.19	0.98–1.44
Model 2	1.94	1.53–2.46	1.71	1.14–2.56	0.80	0.63–1.00

*Note*: Model 1: Unadjusted; Model 2: Adjusted for age, gender, BMI, living alone, education level, IMD, number of health conditions, severity of pain, mobility limitations.

Abbreviations: BMI, Body Mass Index; CI, confidence interval; IMD, Index of Multiple Deprivation; MSK, musculoskeletal; OR, odds ratio.

### 3.6 Association between regional MSK pain and social factors

Participants who reported pain in one or two regions were more likely to report loneliness than those without pain, and this effect remained after adjusting for demographic factors, pain severity and mobility limitations (one painful region vs. none [OR: 1.55; 95% CI: 1.25–1.92]; two painful regions versus none [OR: 1.56; 95% CI: 1.25–1.94]; Table 4). Among participants who reported regional pain, those who reported spinal pain only were most likely to report loneliness (OR 1.88; 95% CI 1.45–2.44; Table A4). Participants who reported pain in two regions were also more likely to report perceived insufficiency of social support (pain in two regions vs. none [OR 1.84; 95% CI 1.26–2.68]). Of those reporting regional pain, those who reported both lower limb and spinal pain had the highest odds of reporting insufficient social support (OR: 2.07; 95% CI 1.36–3.16).

### 3.7 Association between widespread MSK pain presence and social factors

Widespread pain was associated with increased risk of loneliness compared to no pain in unadjusted (OR: 2.83; 95% CI: 2.33–3.45) and adjusted models (OR: 1.94; 95% CI: 1.53–2.46; Table 4). Participants experiencing widespread pain also had increased risk of reporting insufficient social support (OR: 2.63; 95% CI: 1.84–3.77), and this effect remained after adjusting for demographic factors, pain severity and mobility limitations (OR: 1.71; 95% CI: 1.14–2.54). We found no significant association between widespread MSK pain and extent of self‐reported social engagement.

## 4 DISCUSSION

This is a large‐scale cohort study of community‐dwelling older adults and our analysis provides novel insights into the patterns of MSK pain, and the association between pain and social functions among older adults. Overall, MSK pain prevalence was high, with 84.2% reporting pain at one or more bodily site in the past 6 weeks, and 26.1% reporting widespread pain. These respondents reporting widespread pain were more likely to be women, live alone, live in socially deprived areas and have three or more health conditions compared to those who reported no MSK pain. Our findings indicate that individuals who report any MSK pain are more likely to report loneliness or perceived insufficiency of social support compared to those who reported no pain. Individuals who reported widespread MSK pain were most likely to report loneliness or lack of social support. We found no association between any MSK pain, regional or widespread MSK with extent of social engagement.

These data are consistent with other national and international cohorts. The overall prevalence of any MSK pain (84.2%) was similar to that observed in a large Norwegian cohort that used the same pain questionnaire (87% of a sample of 20 to 60 year olds; Kamaleri et al., 2008). Several studies have identified that women are more likely to report widespread pain, and pain across more than one bodily region compared to men, particularly in older age groups (Butera et al., 2019; Leveille, Zhang, McMullen, Kelly‐Hayes, & Felson, 2005; Peat, Thomas, Wilkie, & Croft, 2006). Those who live in areas of higher social deprivation, and report poorer overall health are also at increased risk of disabling MSK pain (Jordan, Thomas, Peat, Wilkie, & Croft, 2008; Leveille et al., 2005).

Loneliness and perceived adequacy of social support were negatively impacted by the presence of any MSK pain, yet social engagement was not. This may be due to the difference in how we measure these constructs. Whilst we measured social engagement by membership of community groups, loneliness and social support are self‐perceived. This finding suggests that while older adults who experience MSK pain may remain socially engaged, they perceive themselves as being less well connected and supported. Loneliness has many detrimental physical, biological and psychological impacts among older adults, and has been recognised as a serious social and public health problem among the ageing population (Shankar et al., 2011). Those involved with the care and management of older adults with MSK pain should consider how self‐perceived loneliness and lack of social support could be enhanced through psychological interventions and support (Cruwys et al., 2014).

The link between MSK pain and loneliness has been examined in several other large‐scale studies. A cross‐sectional analysis of the English Longitudinal Study of Ageing (*n* = 9299; mean age: 65.8 years) found that individuals with MSK pain were more likely to report loneliness (OR: 1.15; 95% CI: 1.01–1.31) but were at lower risk of social isolation (OR: 0.87; 95% CI: 0.75–0.99; Smith et al., 2019). In a study from the United States, Emerson and colleagues explored pain as a risk for loneliness among 1563 older adults over a 4‐year period, reporting that the odds of loneliness onset was 1.58 (95% CI: 1.08–2.32) times higher for those reporting pain at both time points, compared with those who reported no pain at baseline and follow‐up, after controlling for other covariates (Emerson et al., 2018). They suggested that the relationship between pain and loneliness can be bidirectional. Such a link suggests that appropriate pain interventions could help prevent future loneliness, which in turn could prevent a negative cycle of decline and disability (Emerson et al., 2018).

Qualitative studies have also provided important insight into the impact of MSK pain on social factors among older adults. Social withdrawal and social isolation are commonly highlighted in interviews with older people experiencing MSK pain (Lansbury, 2000; Sofaer et al., 2005; Sofaer‐Bennett et al., 2007). Among 93 community‐dwelling older adults from the USA who were interviewed, most participants discussed how their back pain restricted their social life, and impacted upon relationships with their friends and family, leading to experiences of isolation and inability to pursue hobbies (Makris et al., 2017).

Previous studies examining MSK pain have focused on the number of painful sites, rather than the location of sites, or combinations of painful locations, as a predictor of outcomes (Butera et al., 2019). Pain across different regions of the body is likely to differentially impact on outcomes compared to multiple sites of pain within one region. Treatment targeting a single region of MSK pain may have limited benefit. A biopsychosocial approach should be adopted, and interventions should aim to find a balance between targeting specific MSK pain sites and taking a holistic, whole body approach. Incorporating cognitive behaviour principles including increasing self‐efficacy, active choices, goal setting and positive reinforcement may be beneficial for pain management and preventing loneliness, inability to cope and perceived lack of social support from impacting on these individuals' lives (Cruwys et al., 2014; Main & George, 2011). Clinicians should also consider referral for psychological therapies for older people living with MSK pain.

This study has several limitations. The cross‐sectional nature of the data precludes assessment of causality between MSK pain and loneliness, social support and social engagement. Differences were observed between included participants and those excluded from analyses due to missing data. These differences may impact the generalisability of the results. Data were obtained from a self‐reported questionnaire, which may be influenced by misunderstanding or recall. Single questions were used to assess loneliness, social support, social engagement and MSK pain which may introduce bias. Our questionnaire items for MSK pain sites did not specify right or left side of the body, precluding assessment of the presence and impact of bilateral versus unilateral pain as per recommended by ACR for widespread pain. Finally, while the cohort has broad participation across England, the results may not be generalisable to other countries.

Our study highlights areas for future research. These findings provide some evidence of associations between the presence of widespread MSK pain and social limitations. Future studies evaluating these associations in a longitudinal design are needed to determine the nature of these effects over time. We intend to further explore these relationships in future waves of OPAL follow‐up. The causes of social limitations among older adults are multifactorial, and studies characterizing these complex relationships would be valuable. Randomised controlled trials of interventions targeting both MSK pain and social limitations are also needed.

## 5 CONCLUSION

In conclusion, experiencing regional and widespread MSK pain is common among older adults and is negatively associated with loneliness and social support. This finding highlights the importance of identifying and considering pain in multiple anatomical regions, and the impact on social limitations, when managing older adults with MSK pain.

## CONFLICT OF INTEREST STATEMENT

The authors declare no conflict of interests in relation to this work.

## ETHICS STATEMENT

NRES committee London ‐ Brent (REC number 16/LO/0348).

## OPAL STUDY TEAM

Arden N, Barker K, Bruce J, Collins G, Conway O, Darton F, Dutton S, Fairbank J, Fitch J, French D, Garrett A, Griffiths F, Hagan D, Hanson Z, Haywood D, Hewitt A, Hutchinson C, Lamb S, Mallen C, Marian I, Morris A, Nevay L, Nicolson P, Petrou S, Sanchez M, Slark M, Vadher K, Ward L, Watson M, Williamson E.

## OPAL GENERAL PRACTICE TEAM

Alconbury and Brampton Surgeries, Cambridgeshire; Brigstock and South Norwood Partnership, Croydon; Brownlow Group Practice, Liverpool; Buckden and Little Paxton Surgery, Cambridgeshire; Burbury Medical Centre, Birmingham; Civic Medical Centre, Wirral; Cotswold Medical Practice, Cheltenham; Craven Road Medical Centre, Leeds; Cromwell Place Surgery, Cambridgeshire; Eversley Medical Centre, Croydon; Gate Medical Centre, Birmingham; Gosford Hill Medical Centre, Oxford; Grange Hill Surgery, Birmingham; Hall Street Medical Centre, St Helens; Hollow Way Medical Centre, Oxford; Ireland Wood Surgery, Leeds; Keynell Covert Surgery, Birmingham; Kingsfield Medical Centre, Birmingham; Newton Surgery, Leeds; Old Exchange Surgery, Cambridgeshire; Portland Practice, Gloucestershire; Priory Fields Surgery, Cambridgeshire, Priory View Medical Centre, Leeds; Queslett Medical Practice, Birmingham; Rendcomb Surgery, Cirencester; River Brook Medical Centre, Birmingham; Summertown Health Centre, Oxford; Temple Cowley Medical Group, Oxford; The Adam Practice, Poole; The Harvey Practice, Dorset; The Key Medical Practice, Oxford; The Wand Medical Centre, Birmingham; Three Chequers Medical Practice, Salisbury; Vauxhall Health Centre, Liverpool; Wareham Surgery, Dorset.

## SUPPORTING NIHR CLINICAL RESEARCH NETWORKS

Eastern, North West Coast, South London, Thames Valley and South Midlands, Wessex, West of England, West Midlands, Yorkshire and the Humber.

## AUTHOR CONTRIBUTIONS

Sarrah. E Lamb, Esther Williamson and Julie Bruce contributed substantially to the conception and design of the cohort study from which this data is derived. Alana Morris contributed significantly to the acquisition of data. Philippa Nicolson, Esther Williamson, Maria Sanchez‐Santos, Julie Bruce, Alan Silman and Sarrah. E Lamb conceived and designed this analysis and contributed significantly to interpretation of the data. Philippa Nicolson wrote the first draft of this article, and all authors contributed to critical revision for important intellectual content. All authors approved the final version for submission. Sarrah. E Lamb takes overall responsibility for the integrity of the work as a whole.

## Supporting information

Supplementary Material 1
