# Supplementary material for: Musculoskeletal pain and loneliness, social support and social engagement among older adults: Analysis of the Oxford Pain, Activity and Lifestyle cohort
Source: Musculoskeletal Care. 2020 Nov 17;19(3):269–77. doi: 10.1002/msc.1526 (PMC8518502; doi:10.1002/msc.1526)
Supplement: Supplementary file 1 — Supplementary Material 1 [file MSC-19-269-s001.docx]

**Appendix 1:** Characteristics for complete cases included in analysis versus recruited but excluded cases

|  | Complete cases  n=4,977 | Incomplete cases (excluded)  n=432 | p-value |
| --- | --- | --- | --- |
| Age, mean (SD) | 74.7 (6.7) | 77.0 (7.1) | <0.001 |
| Sex, female (%) | 2,540 (51.0) | 244 (56.5) | 0.03 |
| BMI, mean (SD) | 26.6 (4.9) | 26.4 (4.6) | 0.56 |
| Lives alone (%) | 1,408 (28.3) | 147 (36.3) | 0.001 |
| Education level, school only (%) | 3,184 (64.0) | 275 (69.8) | 0.02 |
| IMD, 20% most deprived (%) | 502 (10.8) | 80 (18.5) | <0.001 |
| Number of health conditions |  |  | 0.41 |
| 0 | 854 (17.2) | 66 (15.3) |  |
| 1-2 | 2,971 (59.7) | 256 (59.3) |  |
| ≥3 | 1,152 (23.2) | 110 (25.5) |  |
| Pain severity |  |  | 0.007 |
| No/slight pain | 3,591 (72.2) | 272 (67.2) |  |
| Moderate pain | 1,056 (21.2) | 90 (22.2) |  |
| Severe/extreme pain | 330 (6.6) | 43 (10.6) |  |
| Mobility limitations |  |  | 0.007 |
| No/slight problems walking | 4,034 (81.1) | 308 (76.6) |  |
| Moderate problems walking | 643 (12.9) | 643 (12.9) |  |
| Severe problems walking /Unable to | 300 (6.0) | 300 (6.0) |  |

SD: Standard Deviation; BMI: Body Mass Index; IMD: Index of Multiple Deprivation.

**Appendix 2.** Characteristics of participants (n=4,977) by regional pain presence

|  | No MSK pain  (n=784) | Upper limb pain only  (n=313) | Lower limb pain only  (n=631) | Spinal pain only  (n=536) | Upper limb + spinal pain  (n=425) | Lower limb + spinal pain  (n=617) | Upper limb + lower limb pain  (n=373) | Upper limb + lower limb + spinal pain  (n=1,298) |
| --- | --- | --- | --- | --- | --- | --- | --- | --- |
| Age, mean (SD) years | 74.0 (6.3) | 74.4 (6.5) | 75.0 (6.9) | 74.1 (6.1) | 74.5 (6.6) | 75.2 (7.0) | 74.9 (6.9) | 75.1 (6.9) |
| Sex, female (%) | 333 (42.5) | 149 (47.6) | 279 (44.2) | 238 (44.4) | 232 (54.6) | 313 (50.7) | 198 (53.1) | 798 (61.5) |
| BMI, mean (SD) kg/m² | 25.6 (4.0) | 25.6 (5.1) | 26.8 (4.8) | 25.8 (4.4) | 25.4 (4.3) | 27.1 (5.0) | 27.2 (4.3) | 27.7 (5.5) |
| Living alone, n (%) | 198 (25.3) | 83 (26.5) | 163 (25.8) | 142 (26.5) | 132 (31.1) | 162 (26.3) | 108 (29.0) | 420 (32.4) |
| School education only, n (%) | 489 (62.4) | 193 (61.7) | 386 (61.2) | 321 (59.9) | 263 (61.9) | 396 (64.2) | 250 (67.0) | 886 (68.3) |
| Index of Multiple Deprivation |  |  |  |  |  |  |  |  |
| 5 (20% least deprived) | 302 (38.5) | 102 (32.6) | 219 (34.7) | 194 (36.2) | 154 (36.2) | 228 (37.0) | 130 (34.9) | 409 (31.5) |
| 4 | 165 (21.1) | 82 (26.2) | 142 (22.5) | 129 (24.1) | 87 (20.2) | 123 (19.9) | 73 (19.6) | 279 (21.5) |
| 3 | 153 (19.5) | 90 (28.8) | 140 (22.2) | 109 (20.3) | 86 (20.2) | 125 (20.3) | 80 (21.5) | 284 (21.9) |
| 2 | 85 (10.8) | 19 (6.1) | 70 (11.1) | 68 (12.7) | 55 (12.9) | 88 (14.3) | 48 (12.9) | 157 (12.1) |
| 1 (20% most deprived) | 79 (10.1) | 20 (6.4) | 60 (9.5) | 36 (6.7) | 43 (10.1) | 53 (8.6) | 42 (11.3) | 169 (13.0) |
| No. health conditions |  |  |  |  |  |  |  |  |
| 0 | 285 (36.4) | 69 (22.0) | 114 (18.1) | 146 (27.2) | 68 (16.0) | 77 (12.5) | 39 (10.5) | 56 (4.3) |
| 1-2 | 446 (56.9) | 199 (63.6) | 400 (63.4) | 322 (60.1) | 246 (57.9) | 384 (62.2) | 246 (66.0) | 728 (56.1) |
| ≥3 | 53 (6.8) | 45 (14.4) | 117 (18.5) | 68 (12.7) | 111 (26.1) | 156 (25.3) | 88 (23.6) | 514 (39.6) |
| Pain severity |  |  |  |  |  |  |  |  |
| No/slight pain | 759 (96.8) | 278 (88.8) | 528 (83.7) | 471 (87.9) | 318 (74.8) | 398 (64.5) | 271 (72.7) | 568 (43.8) |
| Moderate pain | 23 (2.9) | 33 (10.5) | 92 (14.6) | 57 (10.6) | 92 (21.7) | 177 (28.7) | 90 (24.1) | 492 (37.9) |
| Severe/extreme pain | 2 (0.3) | 2 (0.6) | 11 (1.7) | 8 (1.5) | 15 (3.5) | 42 (6.8) | 12 (3.2) | 238 (18.3) |
| Mobility limitations |  |  |  |  |  |  |  |  |
| No/slight problems walking | 753 (96.1) | 302 (96.5) | 531 (84.2) | 504 (94.0) | 372 (87.5) | 467 (75.7) | 293 (78.6) | 812 (62.6) |
| Moderate problems walking | 22 (2.8) | 8 (2.6) | 79 (12.5) | 20 (3.7) | 39 (9.2) | 115 (18.6) | 59 (15.8) | 301 (23.2) |
| Severe problems walking/Unable to | 9 (1.2) | 3 (1.0) | 21 (3.3) | 12 (2.2) | 14 (3.3) | 35 (5.7) | 21 (5.6) | 185 (14.3) |

MSK: Musculoskeletal; SD: Standard Deviation; BMI: Body Mass Index; IMD: Index of Multiple Deprivation

**Appendix 3.** Social factor responses of participants by regional pain presence. All values n (%)

|  | No MSK pain  (n=784) | Upper limb pain only  (n=313) | Lower limb pain only  (n=631) | Spinal pain only  (n=536) | Upper limb + spinal pain  (n=425) | Lower limb + spinal pain  (n=617) | Upper limb + lower limb pain  (n=373) | Upper limb + lower limb + spinal pain  (n=1,298) |
| --- | --- | --- | --- | --- | --- | --- | --- | --- |
| Loneliness^†^ | 190 (24.2) | 89 (28.4) | 210 (33.3) | 197 (36.8) | 149 (35.1) | 228 (37.0) | 149 (40.0) | 617 (47.5) |
| Perceived insufficient social support^‡^ | 40 (5.1) | 21 (6.7) | 41 (6.5) | 38 (7.1) | 42 (9.9) | 73 (11.8) | 35 (9.4) | 161 (12.4) |
| Not socially engaged^§^ | 237 (30.2) | 86 (27.5) | 182 (28.8) | 149 (27.8) | 137 (32.2) | 168 (27.2) | 102 (27.4) | 441 (34.0) |

MSK: Musculoskeletal

^†^ Measured using response to “Do you miss having other people around you?”

^‡^ Measured using response to “Do you receive enough support from other people?”

^§^ Measured by membership of: political party, trade union or environmental groups; tenants or residents’ groups or Neighbourhood Watch; church or other religious groups; charitable associations; education, arts or music groups or evening classes; social clubs; sports clubs, gyms, exercise classes, or any other organizations, clubs or societies.

**Appendix 4.** Adjusted^†^ odds ratios (95% CI) for limitations in loneliness, social support and social engagement by pain presence

|  | Loneliness | | Perceived insufficient social support | | Not socially engaged | |
| --- | --- | --- | --- | --- | --- | --- |
|  | OR | 95% CI | OR | 95% CI | OR | 95% CI |
| No MSK pain (n=784) | 1.00 | Reference | 1.00 | Reference | 1.00 | Reference |
| Upper limb pain only (n=313) | 1.19 | 0.86 to 1.64 | 1.34 | 0.78 to 2.34 | 0.89 | 0.66 to 1.21 |
| Lower limb pain only (n=631) | 1.48 | 1.15 to 1.91 | 1.19 | 0.75 to 1.88 | 0.85 | 0.66 to 1.08 |
| Spinal pain only (n=536) | 1.88 | 1.45 to 2.44 | 1.37 | 0.86 to 2.17 | 0.90 | 0.69 to 1.15 |
| Upper limb + spinal pain (n=425) | 1.39 | 1.04 to 1.84 | 1.74 | 1.09 to 2.77 | 1.03 | 0.78 to 1.35 |
| Lower limb + spinal pain (n=617) | 1.56 | 1.20 to 2.02 | 2.07 | 1.36 to 3.16 | 0.79 | 0.64 to 0.99 |
| Upper limb + lower limb pain (n=373) | 1.76 | 1.31 to 2.36 | 1.57 | 0.96 to 2.56 | 0.78 | 0.61 to 1.01 |
| Widespread: Upper limb + lower limb + spinal pain (n=1,298) | 1.94 | 1.53 to 2.46 | 1.71 | 1.14 to 2.56 | 0.79 | 0.63 to 1.00 |

^†^ Adjusted for age, gender, BMI, living alone, education level, IMD, number of health conditions, severity of pain, mobility limitations.

MSK: Musculoskeletal; OR: Odds Ratio; CI: Confidence Interval; BMI: Body Mass Index; IMD: Index of Multiple Deprivation
